# Gram-scale photosynthesis of polyfunctionalized dihydro-2-oxypyrroles using 3DPAFIPN as a halogenated dicyanobenzene-based photosensitizer via a consecutive visible-light-induced electron transfer process

**DOI:** 10.3389/fchem.2024.1407071

**Published:** 2024-08-08

**Authors:** Farzaneh Mohamadpour, Ali Mohammad Amani

**Affiliations:** Department of Medical Nanotechnology, School of Advanced Medical Sciences and Technologies, Shiraz University of Medical Sciences, Shiraz, Iran

**Keywords:** dicyanobenzene-based photosensitizer (3DPAFIPN), polyfunctionalized dihydro-2oxypyrroles, visible-light-induced electron transfer, photosynthesis, renewable energy source

## Abstract

**Background:**

Typically, organic dyes show lower excited state lifetimes, a key hindrance in the development of efficient photoredox processes. Due to their distinctive qualities and efficiency, a particular class of organic chromophores has drawn considerable interest from the scientific community. Thermally activated delayed fluorescence (TADF), is only seen in molecules with a minimal energy gap (usually less than 0.2 eV) between their lowest two excited states, i.e., singlet excited state (S_1_) and triplet excited state (T_1_), is a distinctive property of the molecules under study. Isophthalonitriles are a promising family of chromophores for use as organic photocatalysts because of the ease with which their redox potentials may be adjusted and the prolonged singlet excited states resulting from TADF.

**Methods:**

A sustainable process for the photosynthesis of polyfunctionalized dihydro-2-oxypyrroles has been developed using the Michael-Mannich cyclocondensation of amines, dialkyl acetylenedicarboxylates, and formaldehyde. The development of a green radical synthesis strategy for this family of chemicals is discussed in detail in the current work. This work used a novel halogenated dicyanobenzene-based photosensitizer was used as a photocatalyst. It was dissolved in ethanol, exposed to air at ambient temperature, and triggered by a blue light-emitting diode as a renewable energy source. This project’s main goal is to use a novel conveniently accessible, reasonably priced donor-acceptor (D-A) based on halogenated cyanoarene.

**Findings:**

When exposed to visible light, the 3DPAFIPN [2,4,6-tris(diphenylamino)-5-fluoroisophthalonitrile] photocatalyst, which is a thermally activated delayed fluorescence (TADF), can induce single-electron transfer (SET), providing a simple and green method that is highly effective, energy-efficient, and environmentally friendly. Also, we calculated the turnover number (TON) and turnover frequency (TOF) for polyfunctionalized dihydro-2-oxypyrroles. Gram-scale cyclization has also been shown to be a practical technique for use in industrial applications.

## Introduction

Photoredox catalysis has been used as a starting point for innovative methods in the field of organic chemistry in recent literature (Mohamadpour, 2022a; Mohamadpour, 2022b). Both academia and industries are paying close attention to the topic of photoredox catalysis, which combines metal-promoted reactions with photoredox cycles (Pinosa et al., 2022). To assist the creation of new, potent, and selective metal-promoted reactions, the key area of study involves the use of affordable, easily produced, and effective organic dyes (Gualandi et al., 2021). The widely used inorganic complexes dependent on Ir(III) and Ru(II) must be replaced with organic dyes in this field. These compounds are renowned for their lengthy excited state lifetimes, which may trend toward dynamic quenching when juxtaposed with organic molecules. Typically, organic dyes show lower excited state lifetimes, a key hindrance in the development of efficient photoredox processes. Due to their distinctive qualities and efficiency, a particular class of organic chromophores has drawn considerable interest from the scientific community (Bryden and Zysman-Colman, 2021). Thermally activated delayed fluorescence (TADF), which is only seen in molecules with a minimal energy gap (usually less than 0.2 eV) between their lowest two excited states, i.e., S_1_ and T_1_, is a distinctive property of the molecules under study. The molecules under investigation experience reverse intersystem crossing (RISC) under ambient circumstances, which is assisted by a thermally activated route from the triplet excited state (T_1_) to the singlet excited state (S_1_). A delayed fluorescence phenomenon is produced as a result, which is frequently seen in systems like this. The current aim is to couple the excellent quantum yield of fluorescence with the outstanding efficiency of reduced instruction set computing (RISC). Through the publication of a fundamental (Uoyama et al., 2012), the year 2012 witnessed a substantial contribution to the area of organic light-emitting diodes (OLEDs). The effective use of dicyanobenzene molecules with desired photophysical characteristics and their proven use in OLEDs are both covered in this procedure. Following these original discoveries, comparable TADF chromophores have been used in other fields, including photocatalysis (Yang et al., 2017; Bryden and Zysman-Colman, 2021). Isophthalonitriles are a promising family of chromophores for use as organic photocatalysts because of the ease with which their redox potentials may be adjusted and the prolonged singlet excited states resulting from TADF (Speckmeier et al., 2018). The compound 2,4,6-tris(diphenylamino)-5-fluoroisophthalonitrile (3DPAFIPN) has been extensively employed in several synthetic techniques that are triggered by visible light. Examples of such procedures include intramolecular cyclizations (Flynn et al., 2020; Wu et al., 2020) as well as the creation of C−C (Cardinale et al., 2020; Donabauer et al., 2020), N−C (Zhou et al., 2020), and P−C (Rothfelder et al., 2021) bonds (Pinosa et al., 2022).

Visible light irradiation is regarded as a trustworthy approach for synthesizing organic molecules due to its abundant energy reserves, affordable cost, and the possibility of accessing sustainable energy sources (Mohamadpour, 2021a; Mohamadpour, 2021b; Mohamadpour, 2022c).

**FIGURE 1:**
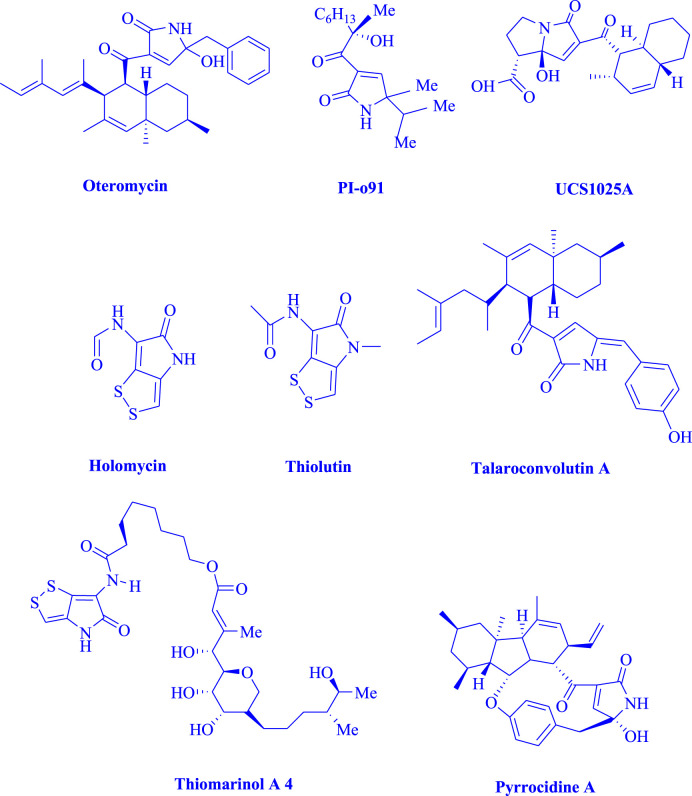
The oxypyrrole rings, which are active pharmaceutically.

Oxypyrrole rings are anticipated to have strong pharmacological and biological properties (Figure 1). HCMV, a protease from the human cytomegalovirus, is one example of pyrrole rings (Borthwick et al., 2002), in addition, human cytosolic carbonic anhydrase isozymes (Alp et al., 2010), PI-091 (Shiraki et al., 1995), Oteromycin (Singh et al., 1995), cardiac cAMP phosphodiestrase (Lampe et al., 1993), and most alkaloids (Chen et al., 2005) are other examples of pyrrole rings.

Several catalysts are used for producing polyfunctionalized dihydro-2-oxypyrroles such as methylene blue (Mohamadpour, 2022d), I_2_ (Khan et al., 2012), glycine (Mohamadpour, 2020), AcOH (Zhu et al., 2009), Cu(OAc)_2_.H_2_O (Lv et al., 2013), Fe_3_O_4_@nano-cellulose–OPO_3_H (Salehi and Mirjalili, 2017), tartaric acid (Mohamadpour et al., 2017), nano-Fe_3_O_4_@SiO_2_/SnCl_4_ (Mirjalili et al., 2019), glutamic acid (Mohamadpour, 2019a), graphene oxide (Bavadi and Niknam, 2018), caffeine (Mohamadpour, 2019b), 2,6-pyridinedicarboxylic acid (Khan et al., 2016), saccharin (Mohamadpour et al., 2016), BiFeO_3_ nanoparticles (Singh and Rajput, 2018), and CoFe_2_O_4_@SiO_2_@IRMOF-3 (Zhang et al., 2017). Intricate processes, long reaction times, the use of expensive reagents, and decreased yields are just a few of the factors that have a big influence on how waste is handled and disposed of. Furthermore, separating homogeneous catalysts from reaction mixtures can be a difficult process. The use of photocatalysts in the creation of organic compounds is discussed in the current studies (Srivastava et al., 2022), with a focus on the adoption of environmentally friendly procedures. According to the inquiry, it is also economically viable to use halogenated organic dye photo-redox catalysts. Using the aforementioned method, a powerful donor-acceptor (D-A) cyanoarene is used as an effective organo-photocatalyst.

Due to its remarkable photophysical and photochemical capabilities, 2,4,6-tris(diphenylamino)-5-fluoroisophthalonitrile (3DPAFIPN) was the main study focus. The variety of photocatalysts available to organic chemists has increased with the introduction of dicyanobenzene-based photosensitizers, which exhibit outstanding photoelectric activity and thermally activated delayed fluorescence (TADF).

A novel halogenated cyanoarene-based donor-acceptor (D-A) photocatalyst; 3DPAFIPN that functions through a series of visible-light-induced electron transfers has been studied in the current work. The process described here uses tandem Michael-Mannich cyclocondensation amines with formaldehyde, dialkyl acetylenedicarboxylates. Additionally, this reaction uses blue LED, a renewable and eco-friendly energy source in an ethanol media as a green solvent, and at room temperature. Regardless of the efficient and prompt fulfillment of all responsibilities and compliance with the agreed financial plan.

## Experimental

### General

A 9100 electrothermal instrument was used to determine the melting points of the different compounds. The DRX-300 Avance equipment from Bruker were used to collect ^1^HNMR spectra with CDCl_3_. Reagents and materials were purchased from Fluka, Merck, and Acros and used immediately.

### The environmentally friendly approach for manufacturing polyfunctionalized dihydro-2-oxypyrroles (5a-s)

Amine **1** (1.0 mmol) and dialkyl acetylenedicarboxylate **2** (1.0 mmol) were agitated for 15 min at room temperature in EtOH (3 mL) with 3DPAFIPN (1 mol%) and blue light (5 W). Then, the reaction mixture was agitated in the presence of amine **3** (1.0 mmol) and formaldehyde **4** (1.5 mmol). Through the use of thin-layer chromatography (TLC), we monitored the progress of the reaction. The unrefined solid was subjected to screening after the chemical reaction, followed by washing with ethanol, eliminating the need for additional purification methods. Current research focuses on whether it is possible to produce the above compounds on a gram-scale for use in pharmaceutical research and development (R&D) processes. 25 mmol of aniline, 25 mmol of n-butylamine, 37.5 mmol of formaldehyde, and 25 mmol of dimethyl acetylenedicarboxylate (DMAD) were used in a single experiment. The 15-min reaction time was followed by the application of a typical filtering technique to recover the end product. A spectroscopic data is included in the Supplementary Material file.

## Results and discussion

**FIGURE 2:**
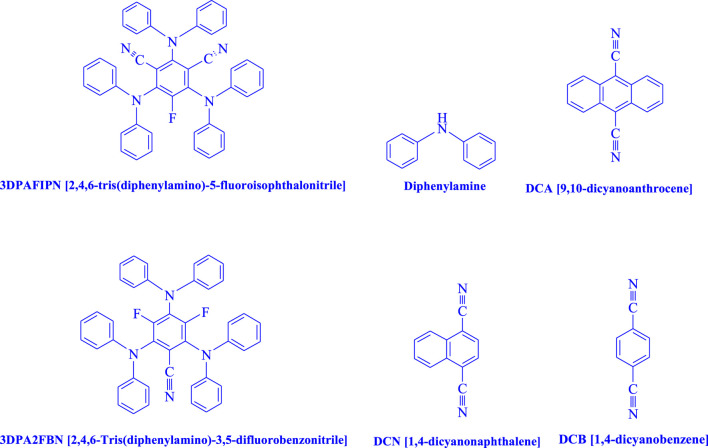
In this investigation, the catalyst’s adequacy was assessed.

**SCHEME 1:**
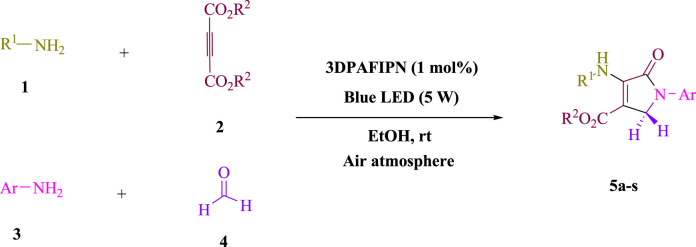
This process presents a green technique for producing polyfunctionalized dihydro-2-oxypyrroles by radical synthesis.

In the current investigation, a 3 mL ethanol medium containing formaldehyde (1.5 mmol), aniline (2 mmol), and dimethyl acetylenedicarboxylate (DMAD) (1.0 mmol) was used to evaluate the reaction. A trace amount of **5c** was generated at room temperature in the presence of 3 mL of EtOH for 45 min without the use of a photocatalyst. Table 1, entry 2, gives a thorough summary of this observation. The addition of photocatalysts accelerated the reaction rate. According to the data shown in Figure 2, the chemicals include 3DPAFIPN, diphenylamine, DCA, 3DPA2FBN, DCN, and DCB. With different yields, **5c** can be produced with the current process. The results presented above indicated an increase in the operational effectiveness of 3DPAFIPN. According to the information in Table 1, entry 1, a reaction with 1 mol% 3DPAFIPN produced a 95% yield. Table 2 shows results for toluene, CH_3_CN, MeOH, EtOH, THF, EtOAc, H_2_O, DCM, CHCl_3_, DMSO, and solvent-free conditions. The reaction was shown to have a noticeably elevated rate and subsequent yield in the presence of EtOH. A yield of 95% was attained based on the information shown in Table 2, notably entry 9. Various light sources have been utilized in research aiming at evaluating the influence of blue light on output, as shown in Table 2. During the evaluation that was carried out without the implementation of an illuminating device, the **5c** was found in a minuscule quantity. The current study shows that the presence of 3DPAFIPN and visible light is a crucial need for the efficient synthesis of product **5c**. Blue light-emitting diode (LED) intensities of 3 W, 5 W, and 7 W were used to find the best designs. According to the study’s findings, it was found that using blue light-emitting diodes (LEDs) with a 5-W power output produced the best outcomes. Table 3 and Scheme 1 show the results of experiments carried out under idealized circumstances on a variety of substrates. The outcome of the reaction was not significantly affected by the addition of an aniline substituent. The substitution of halide functionality in the current reaction was approved. Both reactions involving functional groups that may donate electrons and those involving functional groups that display electron acceptance are permissible in the reaction’s present state. It was discovered that the reactivity shown in aliphatic and benzylic amines was similar. Dimethyl acetylenedicarboxylate (DMAD) and diethyl acetylenedicarboxylate (DEAD) react similarly.

**TABLE 1:** Here is a photocatalyst optimization table for the synthesis of **5c** *^a^*.

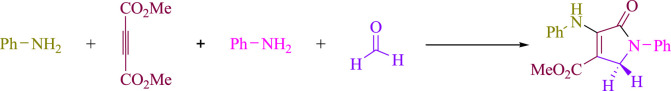				
Entry	Photocatalyst	Solvent (3 mL)	Time (min)	Isolated Yields (%)
1	3DPAFIPN (1 mol%)	EtOH	15	95
2	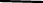	EtOH	45	trace
3	Diphenylamine (1 mol%)	EtOH	15	27
4	DCA (1 mol%)	EtOH	15	23
5	3DPA2FBN (1 mol%)	EtOH	15	80
6	DCN (1 mol%)	EtOH	15	21
7	DCB (1 mol%)	EtOH	15	17
8	3DPAFIPN (0.5 mol%)	EtOH	15	84
9	3DPAFIPN (1.5 mol%)	EtOH	15	95

^*a*^ Reaction conditions: formaldehyde (1.5 mmol), aniline (2 mmol), and dimethyl acetylenedicarboxylate (DMAD) (1 mmol) were mixed with some photocatalysts at room temperature.

**TABLE 2:** The solvent and visible light optimization table for the synthesis of **5c** *^a^*.

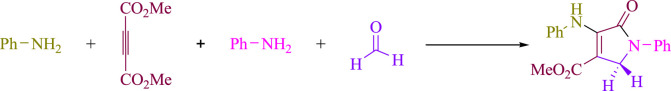				
Entry	Light Source	Solvent (3 mL)	Time (min)	Isolated Yields (%)
1	Green light (5 W)	EtOH	15	88
2	White light (5 W)	EtOH	15	85
3	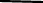	EtOH	45	trace
4	Blue light (5 W)	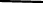	40	39
5	Blue light (5 W)	Toluene	35	34
6	Blue light (5 W)	CH_3_CN	15	79
7	Blue light (5 W)	MeOH	15	71
8	Blue light (3 W)	EtOH	15	90
9	Blue light (5 W)	EtOH	15	95
10	Blue light (7 W)	EtOH	15	95
11	Blue light (5 W)	THF	35	18
12	Blue light (5 W)	EtOAc	15	76
13	Blue light (5 W)	H_2_O	40	33
14	Blue light (5 W)	DCM	45	16
15	Blue light (5 W)	CHCl_3_	45	19
16	Blue light (5 W)	DMSO	30	36

^*a*^ Reaction conditions: formaldehyde (1.5 mmol), aniline (2 mmol), and dimethyl acetylenedicarboxylate (DMAD) (1 mmol) were combined with 1 mol% 3DPAFIPN.

**TABLE 3:** Utilizing the halogenated dicyanobenzene-based photosensitizer; 3DPAFIPN allows for the creation of polyfunctionalized dihydro-2-oxypyrroles.

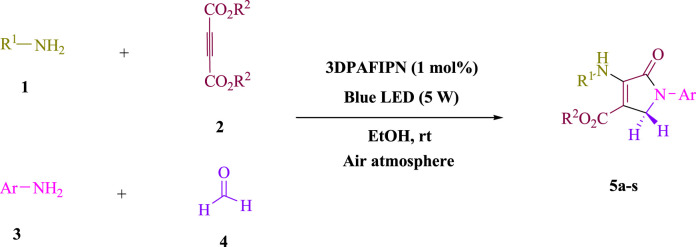	
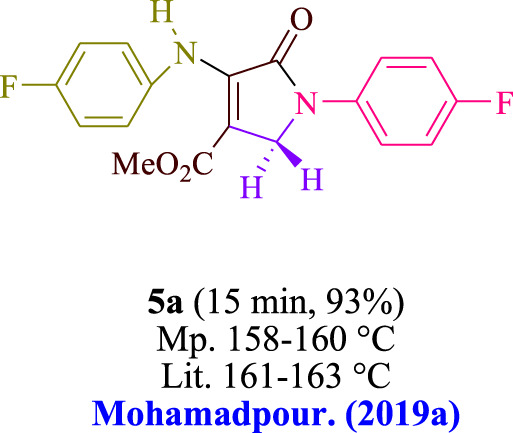	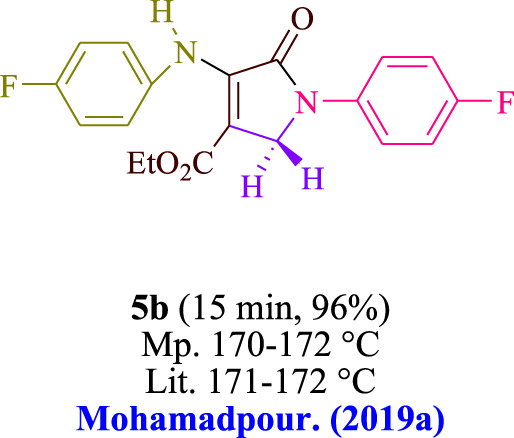
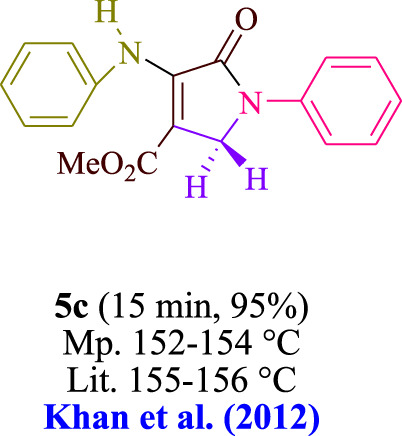	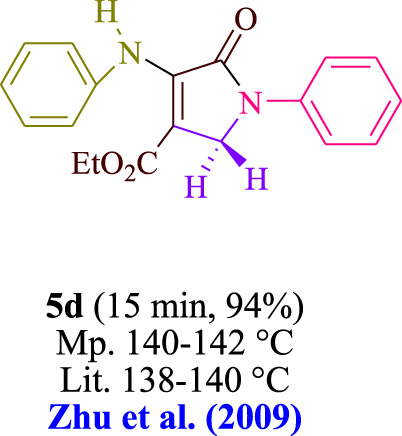
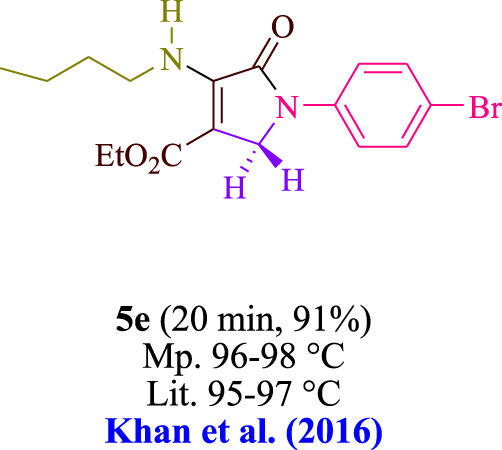	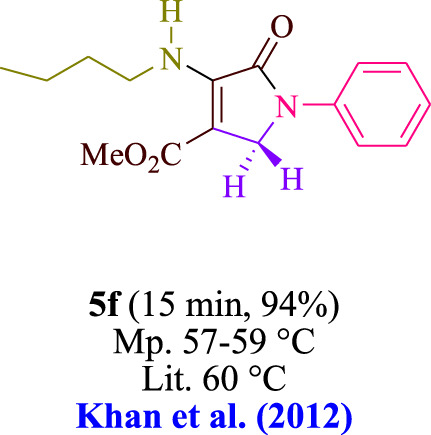
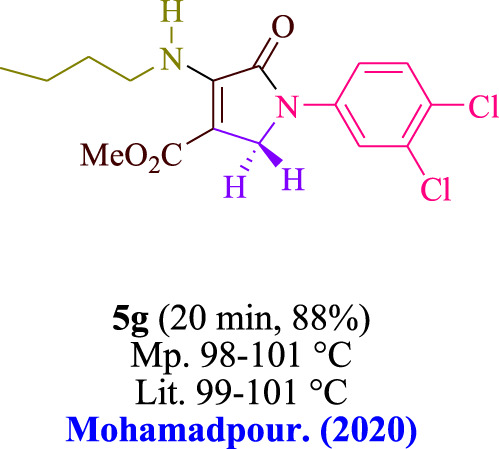	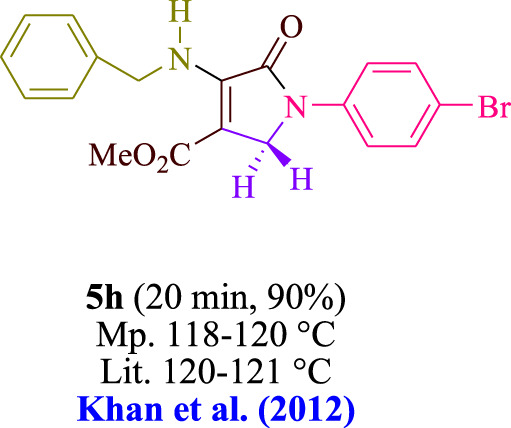
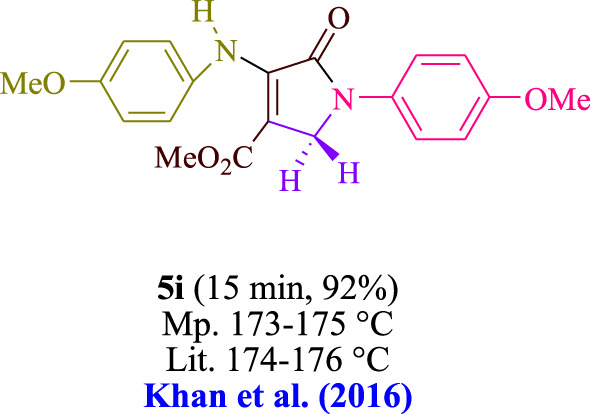	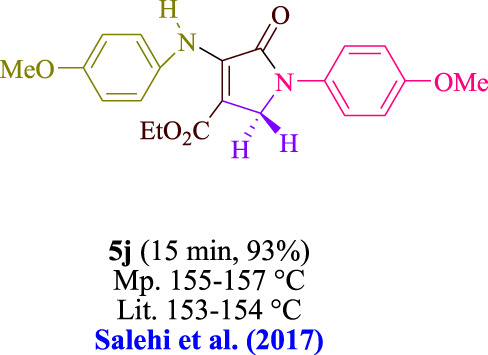
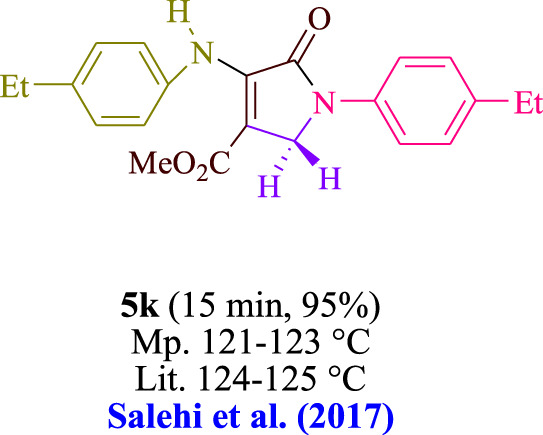	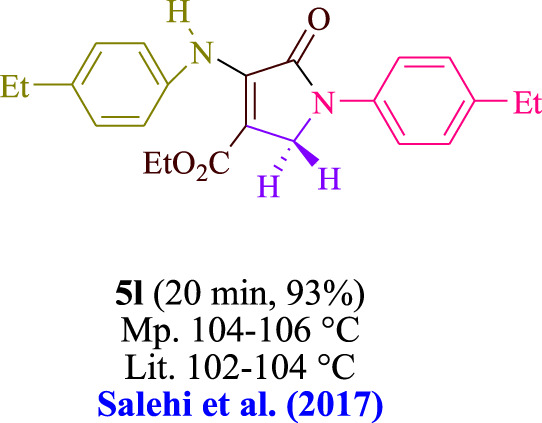
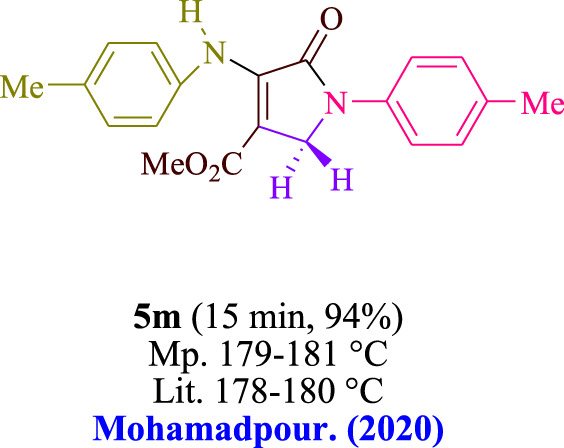	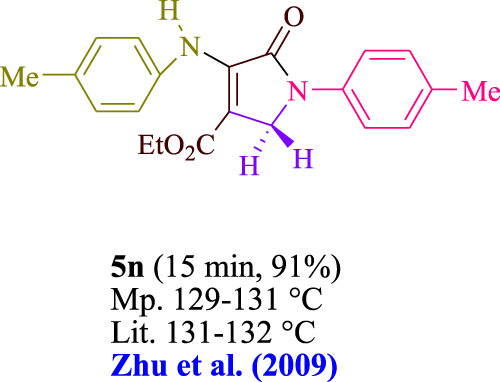
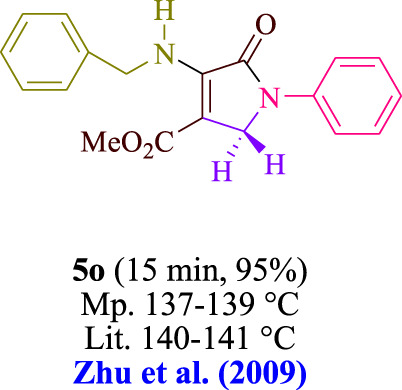	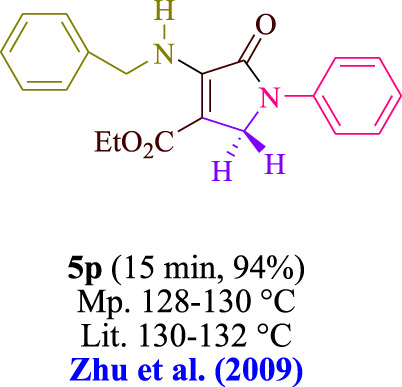
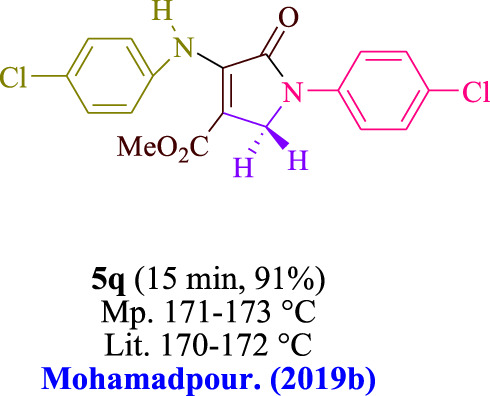	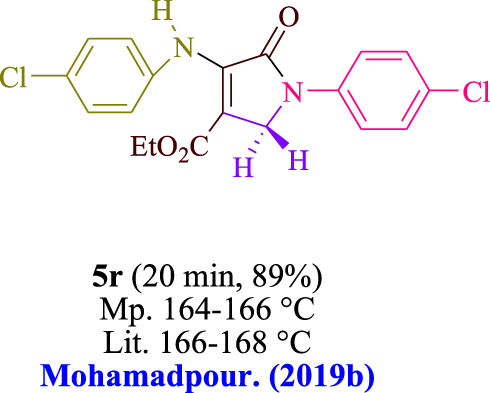
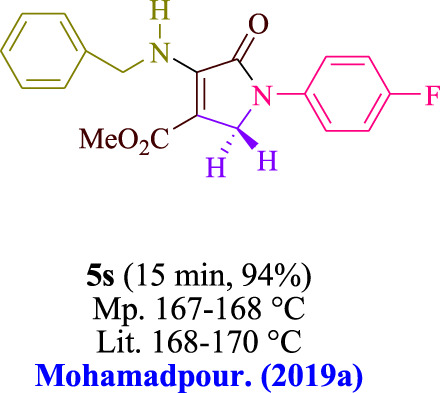	

The turnover number (TON) and turnover frequency (TOF) are presented as objective value measurements in Table 4. In academic literature, the two different forms of yield—Yield/Amount of Catalyst (mol) and Yield/Time/Amount of Catalyst (mol)—are frequently written as TON and TOF, respectively. Increased turnover number (TON) and turnover frequency (TOF) values have the potential to improve catalyst performance since they need less catalyst to provide desired yields. When it comes to **5c**, a TOF of 6.3 and a TON of 95 are both regarded as high. A TOF of 6.2 is considered high concerning **5d**, although a TON of 94 is also considered high. The investigation’s goal was to maximize production, cut down on reaction times, and use the least amount of catalysts possible.

**TABLE 4:** The turnover number (TON) and turnover frequency (TOF) were calculated in the following.

Entry	Product	TON	TOF	Entry	Product	TON	TOF
1	5a	93	6.2	11	5k	95	6.3
2	5b	96	6.4	12	5L	93	4.6
3	5c	95	6.3	13	5m	94	6.2
4	5d	94	6.2	14	5n	91	6
5	5e	91	4.5	15	5o	95	6.3
6	5f	94	6.2	16	5p	94	6.2
7	5g	88	4.4	17	5q	91	6
8	5h	90	4.5	18	5r	89	4.4
9	5i	92	6.1	19	5s	94	6.2
10	5j	93	6.2				

### Control experiments

**SCHEME 2:**
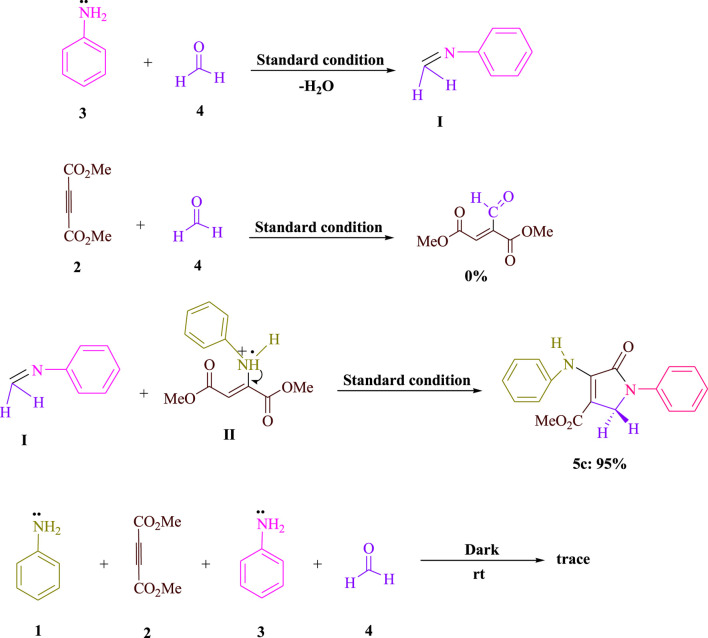
Significant control experiments are done to understand the condensations of formaldehyde (**4**, 1.5 mmol), dimethyl acetylenedicarboxylate (DMAD) (**2**, 1 mmol), and aniline (**1** and **3**, 2 mmol).

**SCHEME 3:**
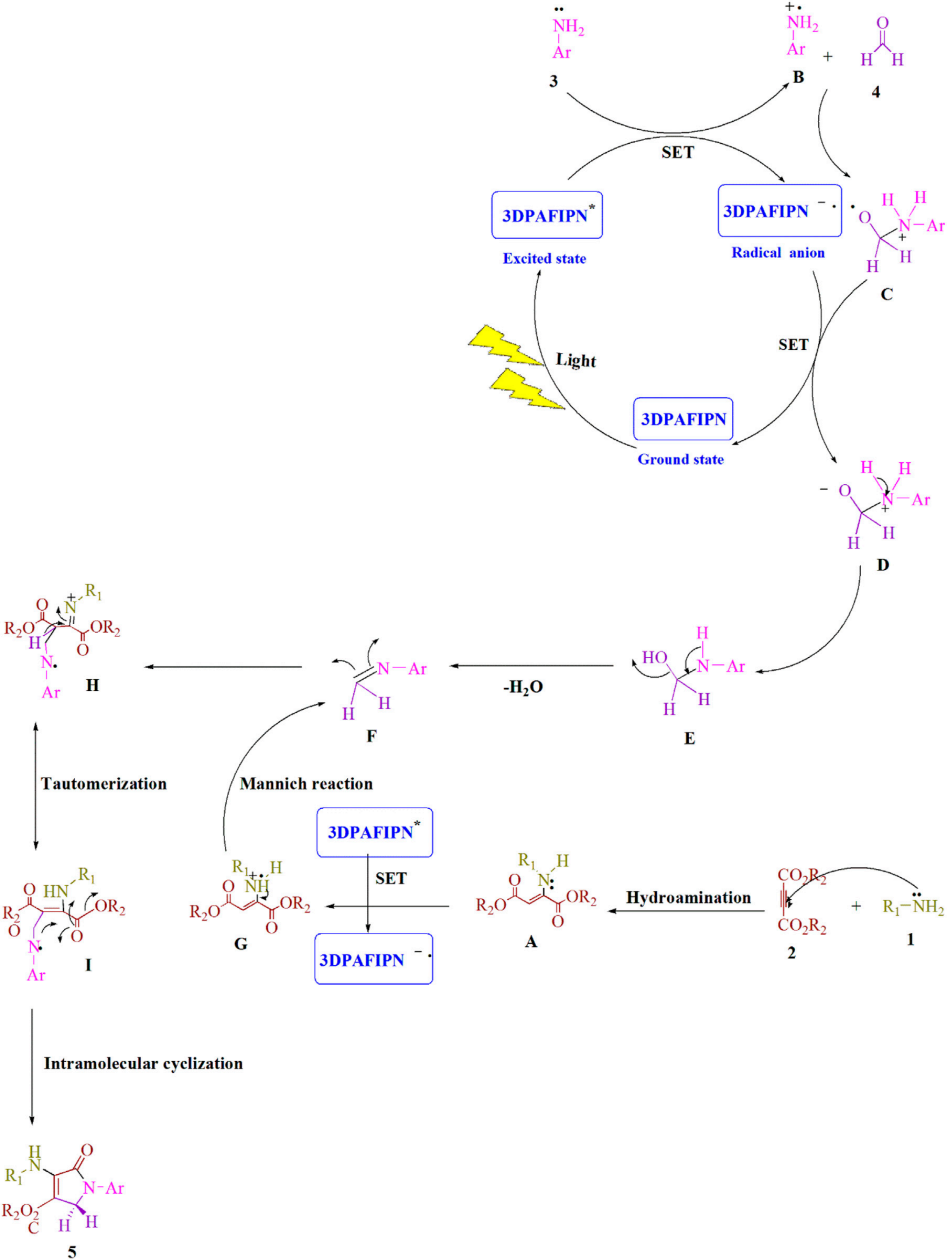
Here, a thorough representation of the synthetic process for producing polyfunctionalized dihydro-2-oxypyrroles is provided.

The results of the control tests carried out to clarify the mechanism using the visible-light-induced are shown in Scheme 2. As shown in Scheme 2, aniline **3**) and formaldehyde **4**) were condensed to produce the related imine **I**) under conventional conditions (3DPAFIPN in EtOH under blue LED). No product was produced when formaldehyde **4**) was mixed with dimethyl acetylenedicarboxylate (DMAD) **2**). The yield for **5c** was 95% for the condensation of imine **I**) and enamine radical **II**). When the reaction was conducted in the dark, a very small amount of the equivalent product **5c** was produced. According to the findings of this experiment, Scheme 3 depicts a logical and cogent chemical progression.

## The suggested mechanism

A detailed explanation of the recommended technique is shown in Scheme 3. The cyanoarene organic dye 3DPAFIPN has been used to create photocatalytic reactions that use visible light energy as a sustainable resource by employing single-electron transfer (SET) processes. Visible light is used to speed up the process. Amine **1**) and dialkylacetylenedicarboxylate **2**) are combined in the Michael reaction to produce enamine **A**). The aniline radical **B**) is created using the SET procedure and visible light irradiation to enhance the visible-light-induced 3DPAFIPN^*^. The result of the reaction between the radical cation **B**) and formaldehyde **4**) is the radical cation **C**). The radical adduct **C**) and the 3DPAFIPN radical produce the intermediate **D**) and the ground state of 3DPAFIPN by a single-electron transfer (SET) mechanism. The intermediate **F**) is created when one H_2_O molecule from **E**) is removed. The SET method is used to create the enamine radical **G**) to improve visible-light-induced 3DPAFIPN^*^. A more stable tautomeric form **I**) is created via the Mannich reaction between an activated imine **F**) and an enamine radical **G**). Finally, a polyfunctionalized dihydro-2-oxypyrrole **5**) is produced by intramolecular cyclization in intermediate **I**).

A comparison of the effectiveness of several catalysts in promoting the production of polyfunctionalized dihydro-2-oxypyrroles is shown in Table 5. The method in issue uses minuscule amounts of photocatalyst and precipitates quick chemical changes without producing any leftover materials. This method can be applied in situations where there are measurable light wavelengths. Atom-economical procedures are very effective and have a significant impact on the industrial domain at multigram scales.

**TABLE 5:** The outcome of evaluating the catalytic effectiveness of the numerous catalysts for the production of **5c** and **5d** *^aysa^*.

Entry	Product	Catalyst	Conditions	Time/Yield (%)	TON	TOF	References
1	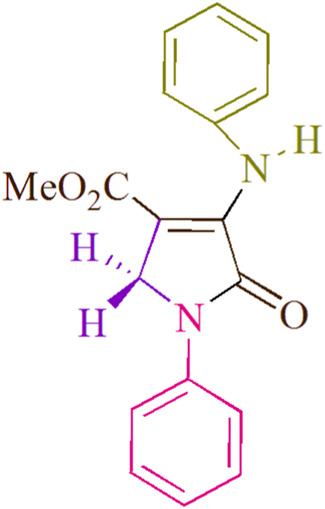	I_2_	MeOH, rt	1 h/82	8.2	0.13	Khan et al. (2012)
2		glycine	MeOH, rt	3 h/93	9.3	0.05	Mohamadpour. (2020)
3		glutamic acid	MeOH, rt	2 h/91	4.5	0.03	Mohamadpour. (2019a)
4		2,6-pyridinedicarboxylic acid	MeOH, rt	1 h/85	8.5	0.14	Khan et al. (2016)
5		3DPAFIPN	blue LED, EtOH, rt	15 min/95	95	6.3	This work
6	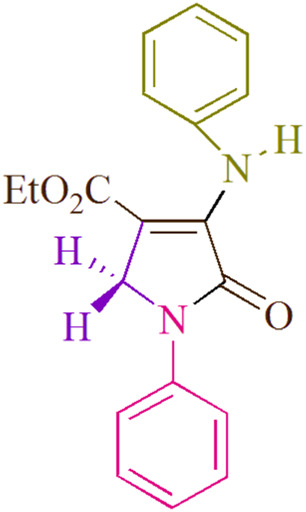	I_2_	MeOH, rt	1 h/81	8.1	0.13	Khan et al. (2012)
7		glycine	MeOH, rt	3 h/90	9	0.05	Mohamadpour. (2020)
8		glutamic acid	MeOH, rt	2 h/88	4.4	0.03	Mohamadpour. (2019a)
9		2,6-pyridinedicarboxylic acid	MeOH, rt	2 h/81	8.1	0.06	Khan et al. (2016)
10		3DPAFIPN	blue LED, EtOH, rt	15 min/94	94	6.2	This work

^*a*^ Aniline, dimethyl/ethylacetylenedicarboxylate, and formaldehyde are used in the synthesis.

## Conclusion

We have used the radical-induced Michael-Mannich cyclocondensation reaction to green photosynthesize polyfunctionalized dihydro-2-oxypyrroles from amines, dialkyl acetylenedicarboxylates, and formaldehyde. The novel halogenated dicyanobenzene-based photosensitizer; 3DPAFIPN was used in current work as a donor-acceptor (D-A) photocatalyst that functions through a series of visible-light-induced electron transfers. The use of blue-light-emitting diode (LED) technology has been shown to produce a sustainable energy-generating mechanism within an ethanol medium at room temperature and in an air environment. The field of chemical synthesis benefits greatly from the proposed technique. These advantages include quick reaction times, elimination of dangerous solvents, increased product yields, simplified reaction mechanisms, durable conditions, and use of a sustainable energy source. Chromatography is not required for the separation technique. It is conceivable to speed up a multigram-scale reaction of model substrates by maintaining the outcome. Thus, the technique may be adopted within a setting that supports long-term ecological and economic viability.

## Data Availability

The original contributions presented in the study are included in the article/Supplementary Material, further inquiries can be directed to the corresponding authors.
