# Supplementary material for: Gram-scale photosynthesis of polyfunctionalized dihydro-2-oxypyrroles using 3DPAFIPN as a halogenated dicyanobenzene-based photosensitizer via a consecutive visible-light-induced electron transfer process
Source: Front Chem. 2024 Aug 8;12:1407071. doi: 10.3389/fchem.2024.1407071 (PMC11338856; doi:10.3389/fchem.2024.1407071)
Supplement: Supplementary file 1 [file DataSheet1.PDF]

Supporting Information

Gram-scale photosynthesis of polyfunctionalized dihydro-2-oxypyrroles using 3DPAFIPN as a halogenated dicyanobenzene-based photosensitizer via a consecutive visible-light-induced electron transfer process

Farzaneh Mohamadpour[a,*], Ali Mohammad Amani[a,*]

[a]Department of Medical Nanotechnology, School of Advanced Medical Sciences and Technologies, Shiraz University of Medical Sciences, Shiraz, Iran

Corresponding authors email: f_mohamadpour@sums.ac.ir, mohamadpour.f.7@gmail.com; amani_a@sums.ac.ir, aliamani@sums.ac.ir

Methyl 3-(butylamino)-2,5-dihydro-2-oxo-1-phenyl-1Hpyrrole-4-carboxylate (5f)

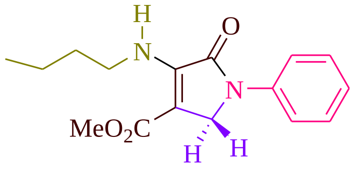

Yield: 94%; m.p. 57-59 °C; ^{1}HNMR (300 MHz, $CDCl_3$): 0.95 (3H, t, *J*= 7.2 Hz, CH_3), 1.45 (2H, sextet, *J*= 7.6 Hz, CH_2), 1.63 (2H, quintet, *J*= 7.6 Hz, CH_2), 3.78 (3H, s, OCH_3), 3.86 (2H, t, *J*=

7.2 Hz, $\underline{CH_2}$–NH), 4.40 (2H, s, $\underline{CH_2}$–N), 6.71 (1H, br s, NH), 7.21-7.28 (1H, m, ArH), 7.51 (2H, d, *J*= 9.6 Hz, ArH), 7.72 (2H, d, *J*= 9.6 Hz, ArH) ppm.

Methyl 3-(benzylamino)-1-(4-fluorophenyl)-2,5-dihydro-2-oxo-1H-pyrrole-4-carboxylate (5s)

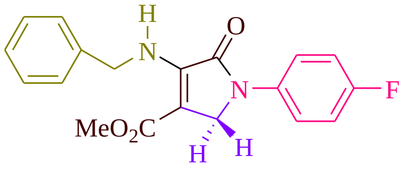

Yield: 94%; m.p. 167-168 °C; ^{1}HNMR (300 MHz, $CDCl_3$): 3.84 (3H, s, OCH_3), 4.42 (2H, s, $\underline{CH_2}$–N), 5.10 (2H, d, *J*= 8.4 Hz, $\underline{CH_2}$–NH), 6.85 (1H, br s, NH), 7.24–7.32 (2H, m, ArH), 7.47-7.53 (5H, m, ArH), 7.75 (2H, d, *J*= 8.4 Hz, ArH) ppm.
